# Sternal Reconstruction Utilizing a Custom 3D-Printed Polyetherketoneketone Implant

**DOI:** 10.1016/j.atssr.2025.09.020

**Published:** 2025-10-21

**Authors:** Eric Lisznyai, Haroutioun Boyajian, Ikenna Okereke, Deangelo Ferguson, Aamir Siddiqui, Andrew M. Popoff

**Affiliations:** 1 Department of Surgery, Henry Ford Health, Detroit, Michigan; 2 Division of Thoracic Surgery, Henry Ford Health, Detroit, Michigan; 3 Division of Plastic and Reconstructive Surgery, Henry Ford Health, Detroit, Michigan

## Abstract

Anterior chest wall reconstruction after sternal resection remains challenging. Implants and prosthetic reconstructive materials should offer ample tissue ingrowth, maintain structural integrity and physiology, minimize infection risk, and maximize cosmesis. Custom titanium implants offer little or no tissue ingrowth and are not modifiable intraoperatively. Reports of polyetheretherketone (PEEK)-based sternal implants cite issues with the above and overall wound healing. Polyetherketoneketone (PEKK)-based implants have been increasingly utilized in odontoid, craniomaxillofacial and vertebral reconstruction. We present a case of sternal resection and reconstruction using a custom printed 3-dimensional PEKK-based implant.

Tumors of the sternum are often categorized based on the extent of resection needed to achieve adequate margins. Smaller tumors may be amenable to limited sternal resection followed by biologic or synthetic coverage to close chest wall defects. However, these coverage methods are limited in their ability to restore adequate chest wall physiology and are prone to wound healing and infectious complications, particularly with larger defects. While 3-dimensional (3D)-printed titanium implants for chest wall reconstruction gained popularity over the past decade, they are relatively inflexible and time-consuming to manufacture.^1^^,^^2^ Polyetheretherketone (PEEK)-based implants offer improved flexibility, but may suffer from increased infection risk and impaired overall wound healing.^3^ Polyetherketoneketone (PEKK)-based implants may offer advantages over both titanium and PEEK implants.

Informed written consent was obtained from the patient to publish the information disclosed in this case report. A 62-year-old active male individual was incidentally found to have a 4.6-cm sternal mass on coronary calcium computed tomography scan (CT) (Figure 1A). Preoperative MRI and CT imaging showed involvement of the sternal body and right chondroid cartilages without evidence of metastatic disease. Biopsy confirmed low grade chondrosarcoma. His case was discussed at a multidisciplinary oncology conference and he was planned for resection and reconstruction.

**Figure 1:**
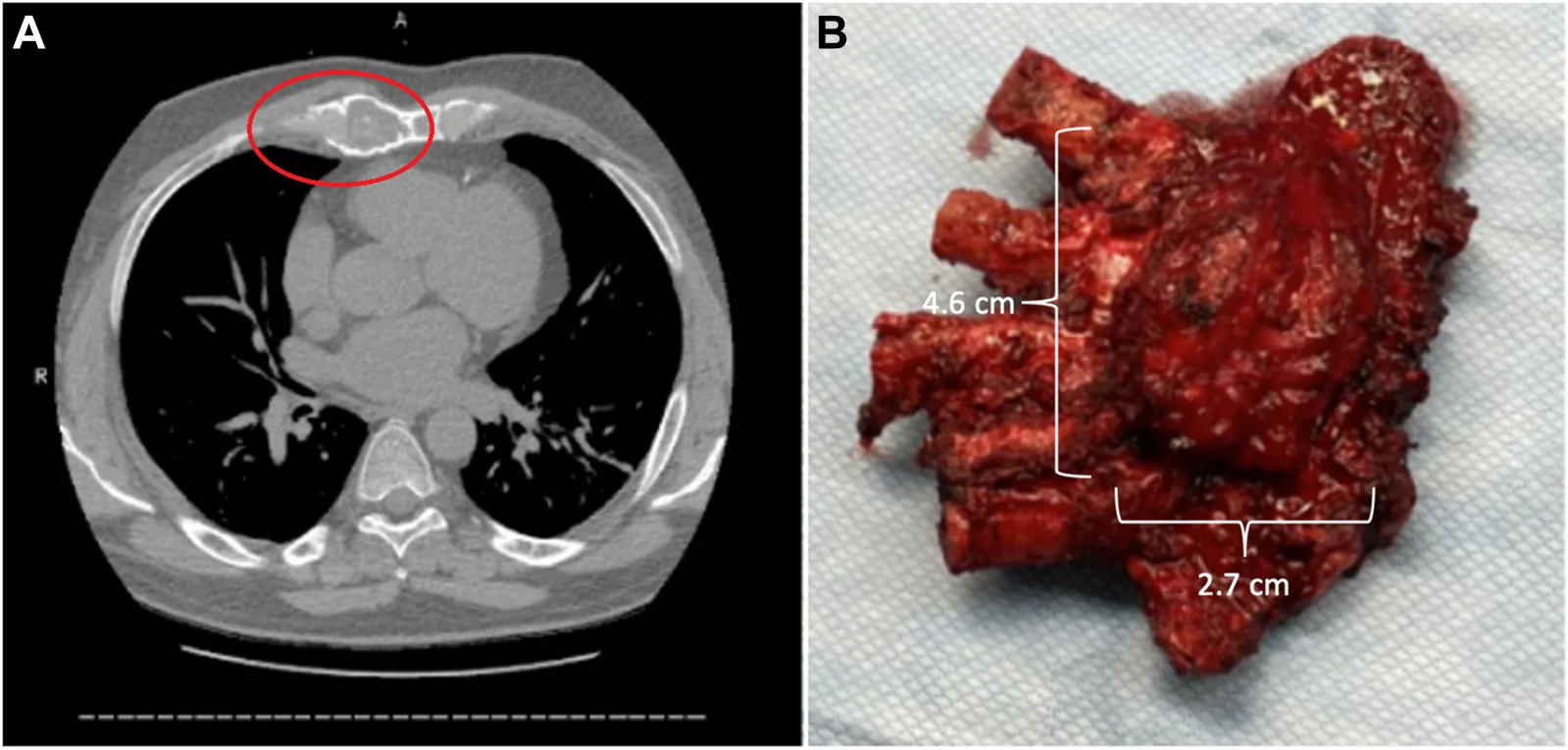
(A) Computed tomography scan showing chest wall mass (red circle). (B) Chondrosarcoma with en bloc resection of sternum and ribs.

A 3D-printed PEKK based sternal implant was designed and manufactured by Oxford Performance Materials using the patient’s CT imaging and modeling software. An anatomical replacement was chosen as the final design, as well as a cutting guide to be used at the time of resection at the sternomanubrial junction. The implant was provided as a custom device under the US Food and Drug Administration Custom Device Exemption Program.

Together, thoracic and plastic reconstructive surgery teams performed an extended total sternectomy with en bloc resection of the sternal body, right chondral cartilages, and overlying portion of pectoralis major with skin including the biopsy site to remove the entire tumor (Figure 1B). Bilateral pectoral flaps were raised overlying the sternum. The manufactured custom PEKK implant was further sized and customized to fit rib extensions with conventional surgical tools. Pilot holes were drilled and the implant was anchored to the manubrium with a ladder plate and screws; it was then secured to the ribs bilaterally using rib plates and screws (Figure 2). The previously mobilized right pectoralis major flap was then rotated for coverage. A left rectus abdominis muscle flap was mobilized and rotated superiorly to ensure healthy, well-perfused tissue overlying the entire implant. The patient’s chest tube, incisional negative pressure dressing, and subcutaneous abdominal drain were removed on postoperative day 3. He was discharged to home on postoperative day 5. Postoperative chest radiograph showed stable implant position (Figure 3A). Final pathology revealed low-grade chondrosarcoma, stage pT1N0M0 with negative margins. The patient has no subjective change in respiratory symptoms and is resuming his active athletic lifestyle. There is no evidence of disease recurrence at 10 months and his incisions are fully healed (Figure 3B).

**Figure 2:**
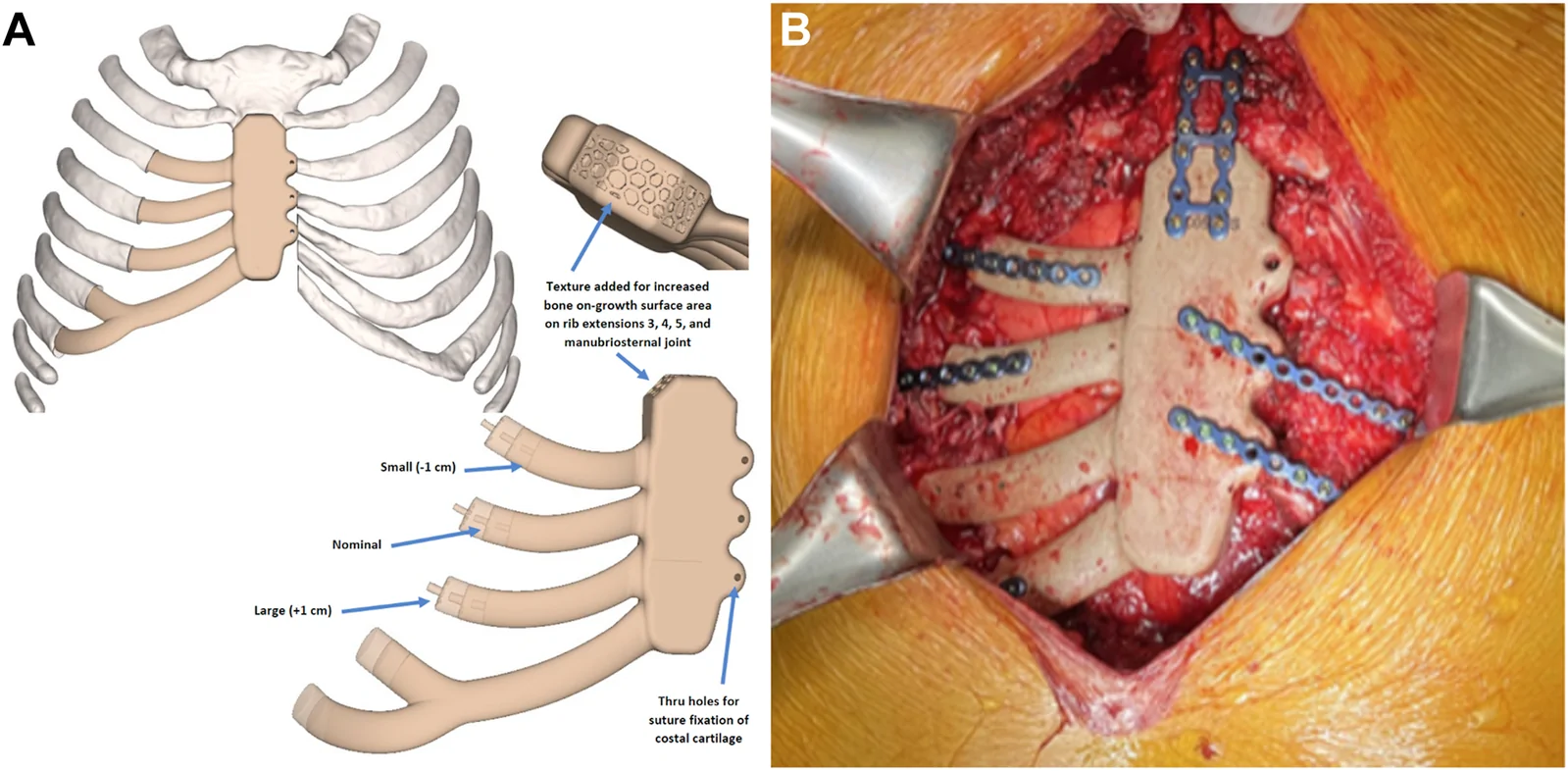
(A) Oxford implant schematics. (Image courtesy of the manufacturer.) (B) Polyetherketoneketone implant after fixation.

**Figure 3:**
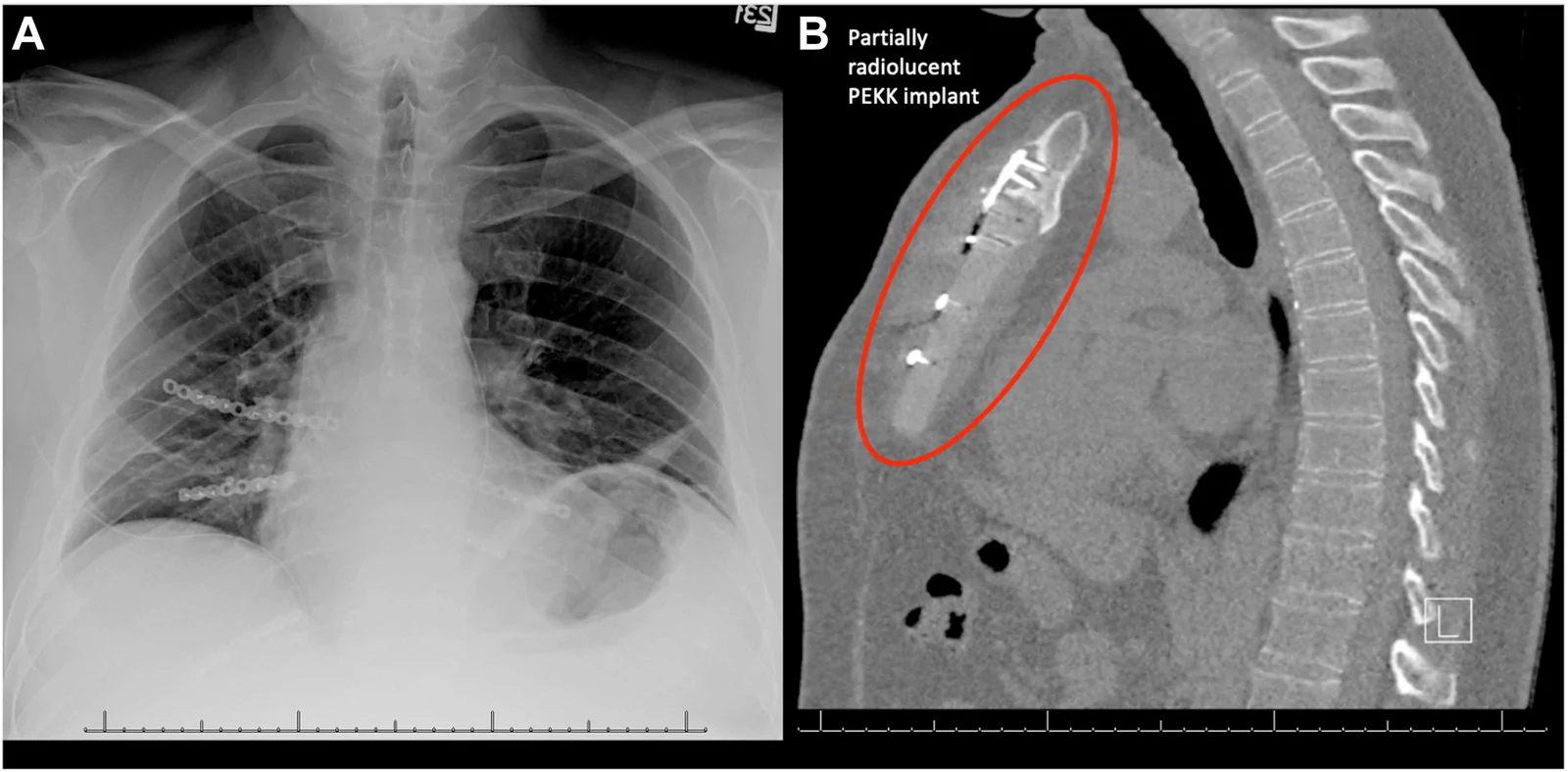
(A) Chest radiograph showing stable implant positioning. (B) Sternoabdominal incision well healed postoperatively (red circle). (PEKK, polyetherketoneketone.)

## Comment

Chondrosarcoma is the most common primary sternal tumor. The mainstay of treatment is radical R0 resection with 2- to 4-cm margins, with or without adjuvant therapy. Overall survival at 5 and 10 years is approximately 60%-70% for low-grade, early-stage tumors. Other favorable prognostic factors include younger age at diagnosis and diameter less than 6 cm.^4^ Overall, these tumors are rare, with 500 cases of primary chest wall tumors per year in the United States.^4^ Resections are often extensive and necessitate planning for both soft tissue and skeletal reconstruction. Protecting underlying mediastinal structures, stabilizing the chest wall, and minimizing chest wall disfigurement are essential elements of the reconstructive process.^4^

While partial sternectomy and reconstruction may be achieved with various nonrigid implants (meshes, biologics, synthetics), extensive sternal defects typically require rigid implant reconstruction. Methylmethacrylate and synthetic meshes were initially utilized, but suffer from ventilatory restriction, high rates of mesh migration, and reported rates of infection as high as 20%.^4^ Titanium implants gained favor given their biocompatibility, lower optical density, very high tensile compressive strength, and high strength-to-weight ratio. However, they offer limited flexibility and tissue integration and can be time-consuming to manufacture, a major drawback for their use in cancer resections.

The advent of custom 3D-printed rigid prostheses has created a more customizable implant process, requiring fewer operative adjustments and allowing for precise fixation to guard against implant migration. Despite this, no consensus exists on the optimum implant material and no large randomized controlled trials exist comparing various materials. Three-dimensional printed titanium implants are now used, but remain susceptible to fracture, displacement, postoperative thoracic pain, and infection.^4^ PEEK sternal implants were more recently introduced with tensile strength approximating that of cortical bone, allowing for improved flexibility. However, these materials are bioinert without natural antimicrobial properties.^1^ PEKK implants offer a unique advantage over PEEK in its ability to promote osteogenesis and its intrinsic bacteriostatic properties. In addition to antibacterial properties, PEKK has manufacturing advantages, as it is faster to print and has more uniform mechanical properties.

In our case, total product cycle time from finalization of design to delivery was approximately 72 hours. This included product printing, finishing, quality assurance, and shipping. Shortened cycle time offers a potential advantage over titanium custom implants, for which this process can take 6-8 weeks or more. Custom 3D-printed titanium implants require surface modifications and lack the ability for intraoperative refinement or additional customization based on the intraoperative findings, extended margin requirements, or other factors. PEKK allows for grinding or filing to shape, drilling, and cutting, offering additional customization options at the time of implantation. In comparing 3D-printed PEKK and PEEK implants, PEKK implants offer a superior surface finish, fully consolidated parts, and higher compressive strength. PEKK also lacks the porosity present in traditional 3D-printed PEEK implants that could contribute to skin irritation and function as infection channels, with greater antibacterial and antiviral effects observed at both 8 and 12 weeks with PEKK.^5^ In addition, the topography of PEKK with peak and pit construction offers superior osseointegration with increased levels of osteoblasts, resulting in accelerated new bone mineralization.^5^ Furthermore, while all 3 materials are approved for use in US Food and Drug Administration–cleared devices, both titanium and PEEK implants are single-level builds requiring mold/scaffold removal prior to use. Titanium and PEEK implants also have fewer processing controls and larger variation in both manufacturers and suppliers.^5^ Boasting a multilevel build, fewer postprocessing steps, and a regulated manufacturing process, the PEKK implant offers a durable, personalized approach to repairing substantial chest wall defects.

In summary, sternal reconstruction can be a challenging problem for thoracic surgeons. PEKK implants offer superior osseointegration to other materials and may have superior antibacterial properties. PEKK implants may also offer reduced postoperative infection risk and an acceptable functional result. Furthermore, PEKK implants have reduced production cycle time compared with both titanium and PEEK implants, are available in various surface finishes including as-printed, polished, and latticed, and allow intraoperative modification. This case report of a novel approach to sternal reconstruction highlights technological advancements, reduced production time, and an excellent functional outcome.
